# YOLOv8-Based System for Nail Capillary Detection on a Single-Board Computer

**DOI:** 10.3390/diagnostics14171843

**Published:** 2024-08-23

**Authors:** Seda Arslan Tuncer, Muhammed Yildirim, Taner Tuncer, Mehmet Kamil Mülayim

**Affiliations:** 1 Faculty of Engineering, Software Engineering, Firat University, 23119 Elazığ, Turkey; satuncer@firat.edu.tr; 2 Faculty of Engineering and Natural Sciences, Computer Engineering, Malatya Turgut Ozal University, 44200 Malatya, Turkey; 3 Faculty of Engineering, Computer Engineering, Firat University, 23119 Elazığ, Turkey; ttuncer@firat.edu.tr; 4 Faculty of Medicine, Kahramanmaras Sutcu Imam University, 46000 Kahramanmaras, Turkey; mkmulayim@ksu.edu.tr

**Keywords:** nail capillary, artificial intelligence, proximal nail fold, YOLOv8

## Abstract

Nail capillaroscopic examination is an inexpensive and easily applicable method to identify capillary morphological changes in patients with conditions such as systemic sclerosis and Raynaud’s. The detection of changes in capillaries makes an important contribution to diagnosing these diseases. Capillary morphology is important in the symptoms of these diseases, and capillary diameter, visibility, distribution, length, microbleeds, blood flow, and density are important indicators in capillaroscopic evaluation. Manual examination to determine these parameters is subjective, causes inconsistent results, and is labor-intensive and time-consuming. To overcome these problems, a YOLOv8s-based system was proposed in this paper to detect the number, thickness, and density of capillaries in the nail bed. The system’s components include database systems that store the analysis results, artificial intelligence-based software that runs on the SBC (Single-Board Computer), and recorded microscope images. mAP and F1_score parameters were used to evaluate the system’s performance, and values of 0.882 and 0.83 were obtained. The proposed system is promising in improving the diagnosis process of diseases such as systemic sclerosis and Raynaud’s by providing objective measurements and the early diagnosis and monitoring of diseases.

## 1. Introduction

Capillaroscopy is the imaging of small vascular structures, known as capillaries, in the nail fold through a microscope. This non-invasive procedure is simple and inexpensive. The main purpose of using this method is to determine whether there is a disease related to capillaries in patients who complain of discoloration of the hands triggered by cold and stress. In patients presenting with this complaint, some changes in the small capillaries in the nail fold help in the early diagnosis of some rheumatic diseases, especially scleroderma. Capillaroscopy provides the opportunity to detect vascular disorders in the early stages and prevent and treat diseases initially. It is easier and more valuable to treat the initial stages of the disease than to treat the chronic stages. Through capillaroscopy, experts can take comprehensive and preventive measures against diseases. Capillaroscopy is frequently used to determine microvascular involvement in systemic diseases such as autoimmune conditions, rheumatism, and many nail and skin diseases [1].

Capillary examination, or capillaroscopy, can be performed with light microscopy and video capillaroscopy, and today, it can also be carried out with dermatoscopy devices. Dermatoscopy devices, which are a fast and effective diagnostic tool for evaluating the nail fold capillary system, are cheap and easy to apply, making these devices advantageous. In practice, there are devices such as Dino-Lite CapillaryScope, Optilia Digital Capillary Scope, and Smart G-Scope capillary scope for imaging purposes [2,3,4]. With the use of these devices, experts diagnose diseases such as scleroderma, Raynaud’s (Reyno), Sjögren’s syndrome, dermatomyositis, rheumatoid arthritis, lupus disease (Lupus rythematosus), diabetes mellitus, and hypertension. It is important to determine the number of capillaries, their thickness, shape, and density per unit area, especially by focusing on the grading of diseases in the images examined.

The quantity of capillaries in one millimeter within the distal row of each finger or toe is known as capillary density. The number and density of blood capillaries are considered common parameters in determining diseases such as scleroderma and Raynaud’s. Karbalaie et al. proposed a new method based on the 90° method for capillaroscopic evaluation. This method was used to evaluate nail fold capillary density [5]. Ingegnoli et al. described nail fold capillary findings using the video capillaroscopy technique in healthy subjects. Nail fold capillaries were examined according to their morphology, size, and density. They reported that the majority of subjects had an average of seven capillaries/mm [6]. Emrani et al. examined a widely used technique for determining capillary density and the connections between capillary count and various grading systems, autoantibodies, pulmonary arterial hypertension, digital ulcers, and scleroderma patterns [7].

Kornaev et al. used high-speed video capillaroscopy to detect and classify capillaries in nail folds. U-Net semantic segmentation method was used for capillary detection. Resnet and Googlenet architectures were used to classify capillaries and 96% accuracy was achieved [8]. Suma et al. examined capillary parameters in the diagnosis of diabetes. Diabetes detection relies on both quantitative and qualitative capillary parameters, including average capillary density, length, breadth, tortuosity, hemorrhages, angiogenesis, and elongated capillaries. They suggested using an object identification method based on deep learning to categorize the nail fold capillaries into five groups: normal, wide, long, tortuous, and bleeding. A total of 600 images were used, and thanks to data augmentation techniques, the number of data used was increased to 1018 [9]. Shah et al. used CNN to determine whether nail fold images obtained using video capillaroscopy could provide diagnostic information about diabetes and its complications. A total of 5236 images were obtained from 120 patients. The area under the ROC curve for five different diabetes mellitus complications was 0.84 [10].

Tello et al. designed an automated software to count nail fold capillaries. In a study using a total of 2713 images, a standard metric precision of 83.84% and a recall of 92.44% were obtained with machine learning algorithms [11]. Natalello et al. evaluated microvascular structure via nail fold video capillaroscopy (NVC) in COVID-19 patients [12]. Bharatti et al. aimed to develop and validate a fully automatic image analysis system. Their proposed method was based on deep learning to detect each capillary in the distal row of capillaries and make morphological measurements. The AUC value obtained was 97% [13]. Korondovych et al. used the Optilia Digital Capilleroscope to distinguish between scleroderma and non-scleroderma capillaries. They analyzed capillary microscopy images with deep learning algorithms, which were equally divided into two groups, including scleroderma and non-scleroderma patterns. A total of 1076 capillaroscope images were divided into training, validation, and test sets, and the same number of images was available in both classes. The accuracy of the model was achieved at 92% [14]. Venkatataphiah et al. proposed a new object detection algorithm based on deep learning architectures to detect and locate various capillary loops in the nail fold region. Various characteristic features were extracted from capillaries through image processing algorithms (YOLOv3), and then discrimination was made between images of diseased subjects and healthy images. In their study, a total of 600 images were analyzed, and the accuracy value was 88.2% [15].

Liu et al. proposed a new deep learning architecture called DAFM-Net for capillary segmentation, as the segmentation of nail fold microbleeds provides valuable pathological information that can lead to further investigation. The network comprised a group normalization layer, dual attention fusion module, and U-shaped backbone. Rich hierarchical representations were generated by the U-shaped backbone, and captured features were used for fine-tuning by the dual attention fusion module. A normalizing technique called group normalization was offered as a helpful way to boost deep neural network convergence. Segmentation tests confirmed the efficacy of the suggested model; the suggested technique, DAFM-Net, demonstrated competitive performance in nail fold microhemorrhage segmentation, with an IOU score of 78.03% and a Dice score of 87.34% in comparison to the ground truth [16]. Hafizh et al. focused on the classification of capillaries. Using the VGG-16 model, they detected nine different data types with an average accuracy of 63.98% [17].

Addou et al. proposed the CNN-based CapillaryNet model. The model is end-to-end and detects capillaries with ~93% accuracy [18]. Nguyen et al. identified capillary types using an improved version of YOLOv5. It is predicted that the system, which yields a mAP50 value of 0.74, will be used in the early diagnosis of diabetes in the future [19]. Yin et al. determined nail fold capillary density. With the improved Yolov5, capillary densities were determined at 85.2% MAP@50 [20]. Nitkunanantharajah et al. imaged the nail sub capillaries of systemic sclerosis patients and healthy controls using optoacoustic imaging and compared them with each other. As a result of deep learning-based classification, 89.7% accuracy was achieved [21].

### 1.1. Motivation

Our main motivation is to automatically detect capillaries in the nail fold and determine the number, density, and thickness of capillaries, which are important in disease diagnosis. The identification of capillaries plays an important role in the diagnosis and follow-up of many diseases, especially scleroderma and Raynaud’s (Reyno). Established guidelines and instructions for the interpretation of capillaries in nail fold images have not yet been standardized. Therefore, the evaluation and interpretation of images are quite subjective.

**Figure 1:**
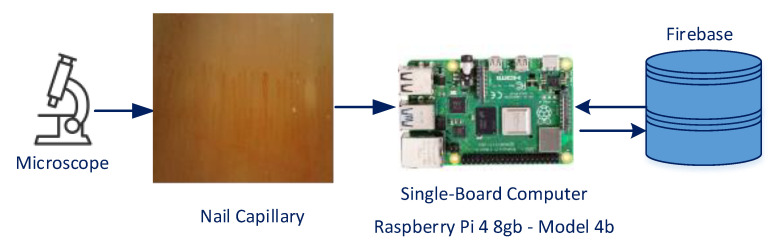
YOLOv8s-based capillary analysis system running on SBC.

The manual identification of capillaries and the determination of their thickness and density are a challenging process. We propose an artificial intelligence-based system to overcome this challenge. The system can automatically detect capillaries in the nail fold and calculate the number, thickness, and density of capillaries, which are important in diagnosing diseases. Figure 1 shows the basic structure of the proposed capillary detection system.

The patient’s nail fold is taken, and the capillaries inside are analyzed using the following four steps:

Step 1: A microscope is used to obtain images of the capillaries.

Step 2: The YOLOv8s architecture trained on the SBC receives the capillary image as input.

Step 3: The capillaries in the image and their thicknesses are measured.

Step 4: The software interface on the SBC displays the analysis findings, which are stored in the database.

### 1.2. Contributions

The following are the study’s primary contributions:

- Thanks to the proposed artificial intelligence-based system, the identification of capillaries in the nail fold becomes automatic.
- Using YOLOv8s, the number, density, and thickness of capillaries are obtained as numerical data.
- This study provides clinical reporting data by overcoming the problems in manual measurements.
- The measurements made are repeatable.

## 2. Data

Capillary thickness and density in the nail bed are important indicators in determining whether a person has a healthy nail bed. While a capillary width of 5–10 μm is considered normal, a capillary width larger or smaller than this value is considered abnormal [22,23]. Average capillary density is the number of capillaries per mm length of the proximal nail fold. The EULAR Study Group on Microcirculation in Rheumatic Diseases defines normal and abnormal capillaries as follows: capillaries with the stereotypical “hairpin” shape, as well as crossing (once or twice) or tortuous capillaries, are defined as “normal”. All other shapes are defined as “abnormal”, provided that the capillary end is convex [22].

**Figure 2:**
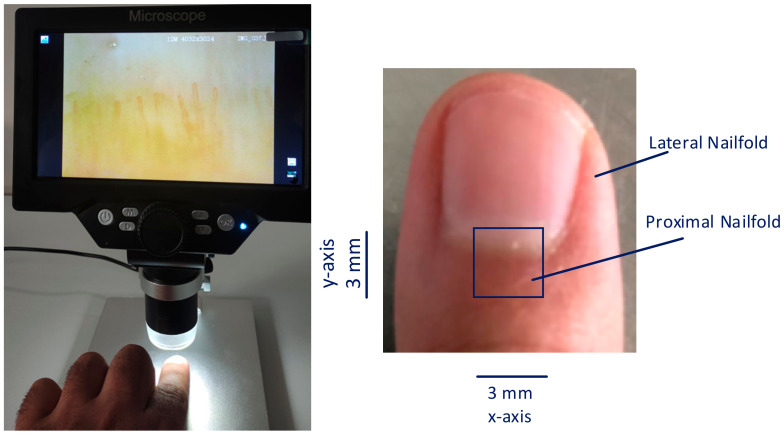
The data collection unit.

The data collection mechanism used in this study is shown in Figure 2. The experimental equipment, MS2 1-1200X 5 Inch 720P LCD Screen USB Digital Microscope, is a 5-inch high-definition LCD screen digital microscope. The lens supports a 1-1200X continuous zoom and solves the problem of high reflection with the help of adjustable-angle LEDs. When connected to a computer via an USB cable, the object can be viewed on the computer monitor and the magnification effect can be displayed on the big screen. The LCD display digital microscope is equipped with a micro-SD card slot. Images obtained during the observation process were stored on a 32 G micro-SD card. The Elazığ Fırat University Ethics Committee approved this study, with approval number 2024/11-36. First of all, a solution that enables the visualization of the superficial structures of the skin and creates a smooth surface was applied to the examined fingers of the patients, and then the capillary image taken from the microscope was recorded.

A total of 800 subjects were identified for obtaining the data. However, capillaries could not be obtained from some of these subjects. Images obtained from 23 subjects were excluded from this study. Nail capillary images of a total of 777 patients were collected. A total of 80% of the data was used for training and 20% for testing. For the data set collected in this study, data were collected regardless of whether the capillaries were normal or abnormal.

**Figure 3:**
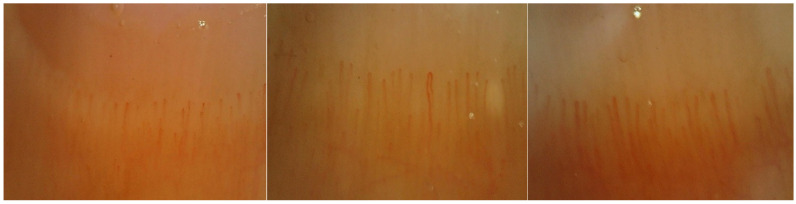
Capillaries in the proximal nail fold.

Each captured image was 3648 × 2736 in size. The collected data were converted to 640 × 640, 96 dpi, so that it could be fed to YOLOv8’s input. Each image was obtained from the proximal nail fold region and this image represented 3 mm horizontally and vertically. Figure 3 shows three different images of capillaries obtained from patients.

**Figure 4:**
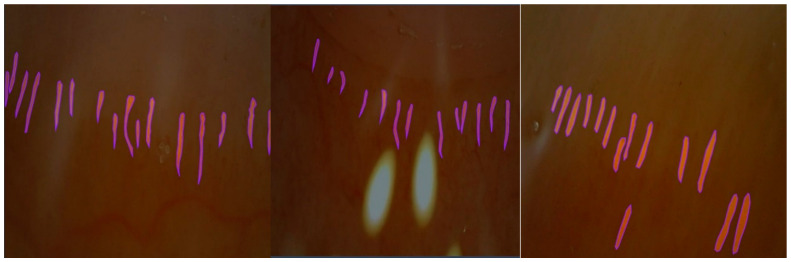
Manually segmented images of capillaries.

To automatically segment the images taken, manual segmentation was first performed by a doctor. The Roboflow environment was used for manual segmentation. Figure 4 shows images with manual segmentation.

## 3. The Proposed Method

**Figure 5:**
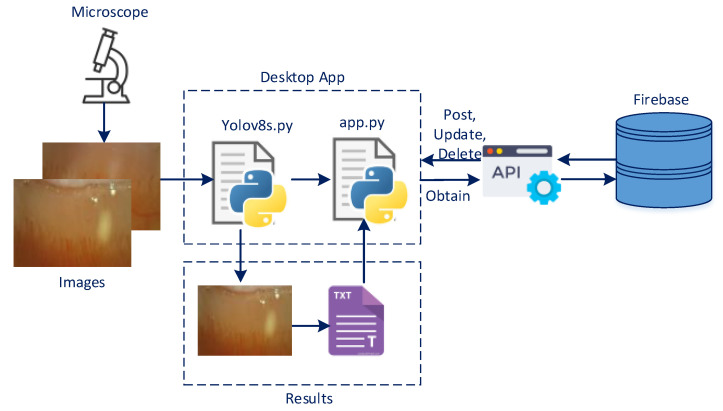
The proposed model.

The YOLOv8s-based analysis system developed in this article to determine the number, width, and density of capillaries in the nail fold is shown in Figure 5. The Python programming language was used to implement the YOLOv8 architecture, which uses images obtained from the microscope as input. YOLOv8s was preferred for use in the this study because it provides faster and more successful results than many object detection algorithms used in real-time object tracking. The system detects possible capillaries in the given image, as well as their number, thickness, and density in the image. It then generates a report containing the results.

**Figure 6:**
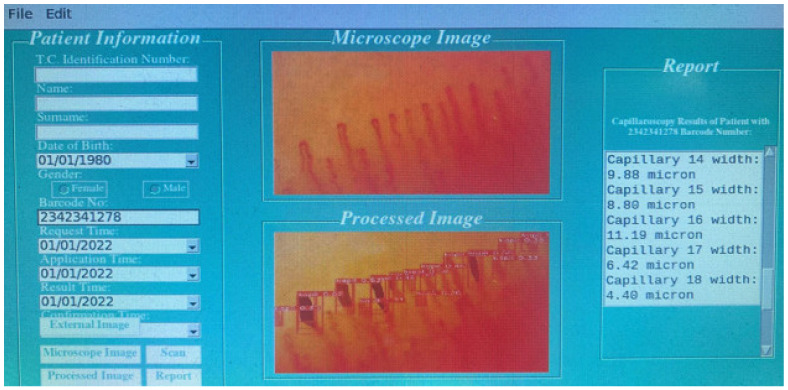
Interface of the proposed system.

Tkinter GUI packages and the Python programming language were used to code the interfaces and integrate the system. The system’s numerical outputs were stored on Firebase, a free platform designed by Google for building web and mobile applications. Figure 6 shows a patient’s information, the nail fold image taken from the patient, the capillaries obtained with YOLOv8s, and the interface related to the report produced by the model.

**Figure 7:**
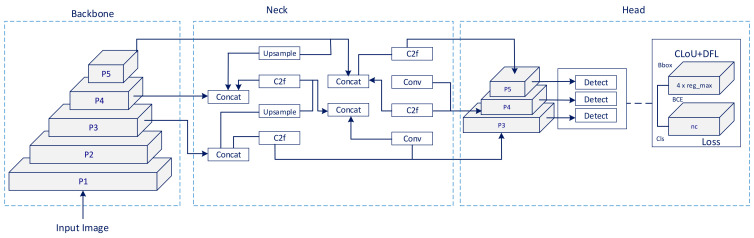
YOLOv8 architecture.

The YOLOv8s architecture used in the article is given in Figure 7. YOLOv8 consists of three main parts: Spine, Neck, and Head [24]. The backbone is responsible for extracting meaningful features from images, the Neck is responsible for feature fusion and integrating contextual information, and the Head is responsible for determining bounding boxes and confidence scores for object detection.

Each bounding box determined in object detection has four coordinate values, (*x*, *y*, *w*, *h*), where (*x*, *y*) represent the center coordinates of the box and (w, h) represent the width and height of the box.

These coordinates are extracted from the network’s output tensor and then inserted into activation functions (usually sigmoid). These values are then normalized to a predetermined grid cell size. The *x* and *y* coordinates are usually positioned relative to the upper left corner of this cell and then proportioned to the grid cell size. The *w* and *h* values are typically multiplied by the width and height of the image, thus expressing it in actual pixels. In the equations below, bx and by represent the center coordinates of the bounding box and bw and bh represent its width and height, respectively. *t_x_*, *t_y_*, *t_w_*, and *t_h_* are the predicted values from the network’s output tensor. cx and cy are the coordinates of the upper left corner of the cell. *p_w_* and *p_h_* are predetermined scale factors.

(1) $$b_{x}=\sigma \left(t_{x}\right)+c_{x}\;b_{y}=\sigma \left(t_{y}\right)+c_{y}\;b_{w}=p_{w}e^{t_{w}}\;b_{h}=p_{h}e^{th}$$

During training, an error function is used to measure how far the model’s predicted bounding boxes are from actual object locations. This error function is the weighted sum of the localization loss, confidence loss, and classification loss components.

(2) $$\begin{array}{ll} Loss= & \lambda_{coord}\sum_{i=0}^{S^{2}}\sum_{j=0}^{B}1_{ij}^{obj}\left[{\left(x_{i}-{\hat{x}}_{i}\right)}^{2}+{\left(y_{i}-{\hat{y}}_{i}\right)}^{2}\right]\\ & +\lambda_{coord}\sum_{i=0}^{S^{2}}\sum_{j=0}^{B}1_{ij}^{obj}\left[{\left(\sqrt{w_{i}}-\sqrt{{\hat{w}}_{i}}\right)}^{2}+{\left(\sqrt{h_{i}}-\sqrt{{\hat{h}}_{i}}\right)}^{2}\right]\\ & +\sum_{i=0}^{S^{2}}\sum_{j=0}^{B}1_{ij}^{obj}{\left(C_{i}-{\hat{C}}_{i}\right)}^{2}\\ & +\lambda_{nonbj}\sum_{i=0}^{S^{2}}\sum_{j=0}^{B}1_{ij}^{obj}{\left(C_{i}-{\hat{C}}_{i}\right)}^{2}\\ & +\sum_{i=0}^{S^{2}}1_{ij}^{obj}\sum_{c\in classes}{\left(p_{i}\left(c\right)-{\hat{p}}_{i}(c)\right)}^{2} \end{array}$$

In this equation, the parameters *λ_coord_* and *λ_noobj_* are weight hyperparameters used to adjust the importance of different components, whereas $1_{ij}^{obj}$ and $1_{ij}^{noobj}$ show which bounding box the cells belong to. The actual bounding box coordinates are *x_i_*, *y_i_*, *w_i_*, and *h_i_*. The estimated bounding box coordinates are ${\hat{x}}_{i}$, ${\hat{y}}_{i}$, ${\hat{w}}_{i}$, and ${\hat{h}}_{i}$. The actual and predicted class parameters *C_i_*, ${\hat{C}}_{i}$, *p_i_*(*c*), and ${\hat{p}}_{i}(c)$ denote the actual and predicted class probabilities.

During training, stochastic gradient descent (SGD) was used to reduce the error function. When SGD is used, the update rule for each weight parameter *θ* is expressed as in Equation (3)

(3) $$\theta =\theta -\alpha \frac{\partial Loss}{\partial \theta }$$

where *α* represents the learning rate and $\frac{\partial Loss}{\partial \theta }$ represents the derivative concerning the weight parameters of the loss function.

## 4. Experimental Results and Discussion

### 4.1. Experimental Results

In this section, the YOLOv8s capillary analysis system is evaluated with the appropriate parameters.

The error matrix is another name for the confusion matrix. The confusion matrix layout visualizes an algorithm’s performance. This is known as a matching matrix, in which an actual class is represented by the column and a predicted class by the row [23]. This matrix contains the fundamental definitions (*TP*, *TN*, *FP*, and *FN*).

True positive (*TP*): The case in which the model predicts the positive class with accuracy.

True negative (*TN*): The case in which the model correctly predicts the negative class.

False positive (*FP*): The case in which the model mispredicts the positive class.

False negative (*FN*): The case in which the model mispredicts the negative class.

We use the following four metrics to evaluate the performance of the YOLOv8s model used in the paper. These are *F*_1_ *score*, recall, precision, and Mean Average Precision (*mAP*).

(4) Precision=TP/(TP+FP)

(5) Recall=TP/(TP+FN)

(6) $$F_{1}\_score=\frac{2\times Precision\times Recall}{Precision+Recall}$$

(7) $$AP=\sum_{k=0}^{k=n-1}\left[Recall\left(k\right)-Recall\left(k+1\right)*Precision(k)\right],\;n=\mathrm{number of thresholds}$$

In object detection, there may be more than one object class (such as capillary background) to be detected. *mAP* calculates the *AP* for each class as in Equation (7) and then calculates the average of these *AP* values. The *mAP* given in Equation (8) provides an overall evaluation of the model’s performance in all classes.

(8) $$mAP=\int_{0}^{1}p\left(c\right)dc$$

**Figure 8:**
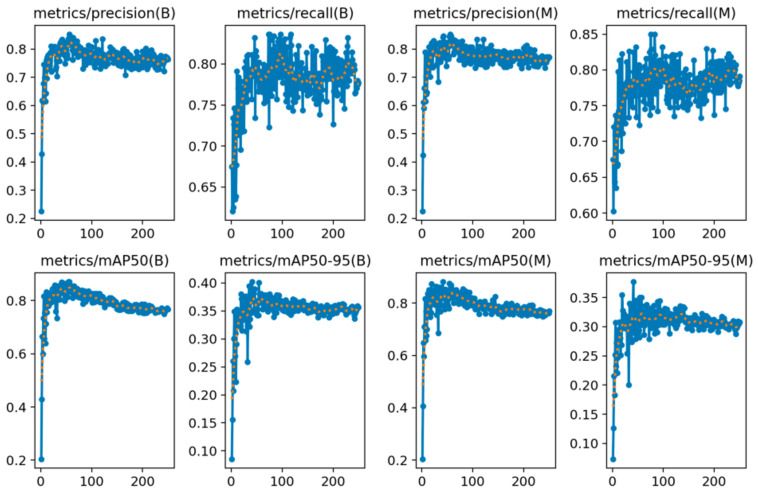
Accuracy changes for YOLOv8s.

The training process was performed in Google Colab. During training, the Google Colab Pro + version was selected. Thus, the highest GPU usage was achieved with the Google Colab Pro+ version. The hyperparameters used for training the model are as in Table 1. Training accuracies for Boxes and Masks are as in Figure 8.

**Figure 9:**
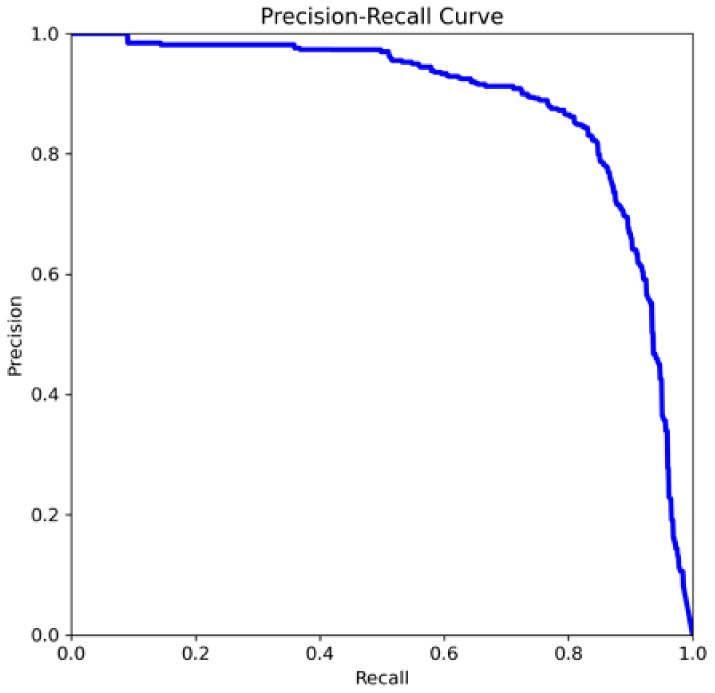
Precision–recall change in the model.

F1_score and mAP50 parameters were obtained as 0.83 and 0.882. The mAP50 value shows that the model is capable of correctly recognizing capillaries. Figure 9 shows that the model has both high precision and high recall variation. The fact that the precision-recall change approaches to the right and above the axis is an indication that the model performs well.

### 4.2. Discussion

**Figure 10:**
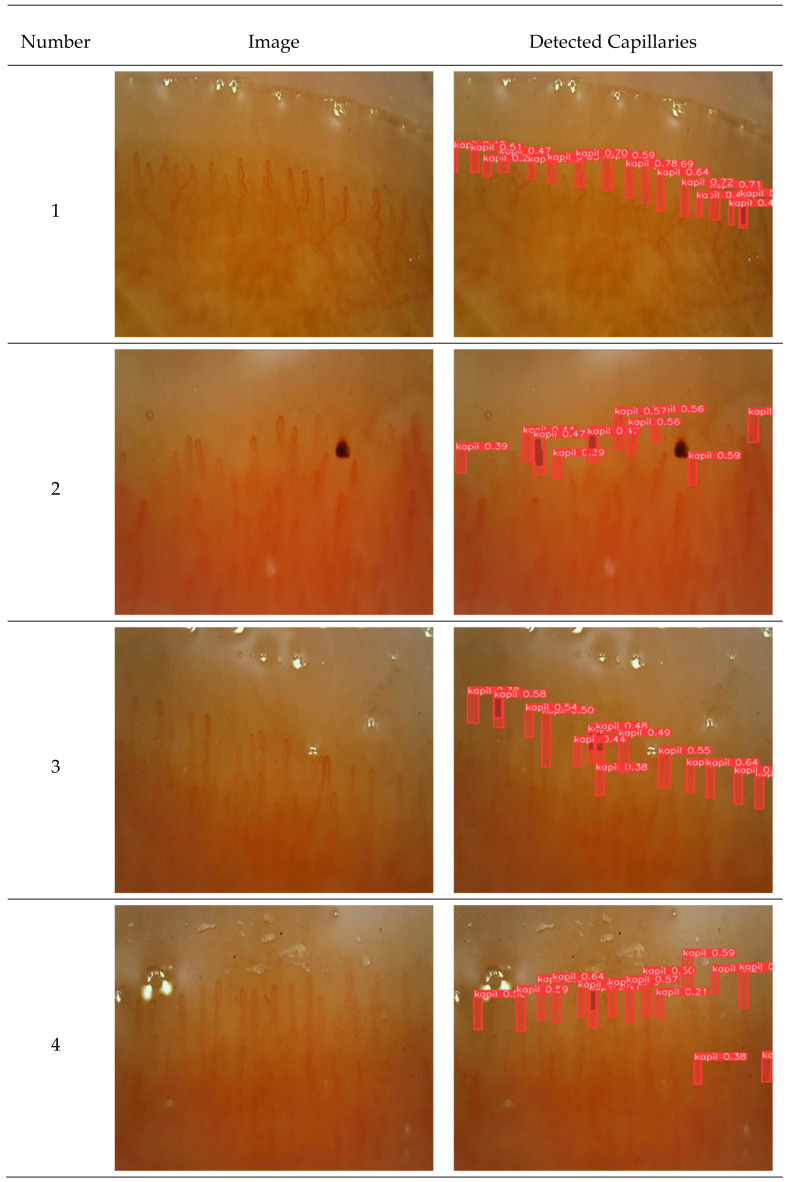
Capillaries detected by the model.

In this study, YoloV8s was used to automatically detect nail fold capillaries. A total of 777 nail fold images with a size of 3648 × 2736 were collected. Each image had a size of 640 × 640 and the horizontal and vertical resolution was 96 dpi. Figure 10 shows the capillary images of different patients and the capillaries detected in these images. The following procedure was applied for the width of each detected capillary.

First, the physical length per pixel was calculated.

The actual size of the image was 3 mm horizontally and vertically.

Since the DPI value was 96, it corresponded to 96 pixels per 1-inch area. The *x*-axis was 3 mm, which is approximately 0.11811 inches in length. Therefore, for 3 mm, there will be approximately 0.11811 × 96 × 640 = 7246.76 pixels. By dividing the actual length of the image by the number of pixels, the size of one pixel is approximately (3 mm/7246.76) 0.0004142 mm, which is 0.4142 microns. Considering that the width of a normal capillary is 5–10 μm, in this study, the width of each capillary is 12 pixels to 24 pixels. For example, in image no. 3, a total of 11 capillaries were detected, and the widths of these capillaries on the horizontal axis are 22, 16, 15, 16, 19, 13, 8, 7, 22, 26, 13 pixels, respectively. The thicknesses of these capillaries in μm are 9.11, 6.63, 6.21, 6.63, 7.87, 5.38, 3.31, 2.9, 9.11, 10.76, 5.38, respectively. According to these results, the thickness of two capillaries was found to be significantly lower than normal, and the thickness of one capillary was determined to be greater than normal.

Table 2 shows the number of capillaries detected, capillary density in 1 mm, and the average capillary thickness for the four images given in Figure 10.

Manual examination takes a long time to determine the number of capillaries and their thickness. Thanks to the proposed method, capillary thicknesses and densities can be determined automatically. The disadvantages of manual measurements are eliminated, and inconsistent results are eliminated by providing an objective evaluation. Thanks to the developed software, it is possible to use the model in high-volume laboratories. Report sharing and reproducible results are now available.

There are no recorded studies in the literature that use YOLOv8 to examine capillary thickness and density in nail fold capillary images. Similar studies based on Yolov3 [15] and Yolov5 [19,20] are available in the literature. However, our work has some fundamental differences from [15,19,20]. The first of these is that our study is an automatic analysis system that combines software and hardware. Another is that it can calculate capillary thickness and capillary density. Our experimental results have shown that the proposed system provides better performance than the approaches in [9,14] in determining the number, thickness, and density of capillaries.

The proposed method also has some limitations. These can be summarized as follows:

- Different types of capillary structures have not been examined.
- The system requires more computing power during the training phase.

## 5. Conclusions

Our work consists of an SBC that processes nail bed images taken by a USB camera and enables the YOLOv8 model to run on it. In this paper, YOLOv8 architecture, one of the state-of-the-art algorithms that can automate the determination of the density and number of nail fold capillaries, was used.

The proposed system not only improves the diagnostic process of diseases such as scleroderma by providing objective measurements but also facilitates the early diagnosis and monitoring of diseases. The integration of YOLOv8s into SBC for the acquisition and analysis of vascular images in the nail bed represents a cost-effective and efficient approach to capillaroscopy. As a result, the system shows promise for wider application in clinical settings, potentially contributing to improving patient outcomes through more accurate and timely diagnosis. The focus of our future research is on further improving the system’s capabilities and considering other conditions affecting capillary morphology.

## Figures and Tables

**Table 1:** The configuration of training parameters for the YOLOv8s models.

Optimizer	SGD
Epochs	250
Batch	16
Image size	640 × 640
Learning rate	0.002
Momentum	0.9
Weight (decay)	0.0005

**Table 2:** Calculated parameters of the detected capillaries.

Image Number	Number of Detected Capillaries	Density	Capillary Thickness Average Micron
1	17	5.67	7.31
2	11	3.67	7.63
3	17	6.67	6.66
4	16	5.34	6.88

## Data Availability

Data will be shared upon request.
